# Exploring the implications of modified advanced lung cancer inflammation index on outcomes in patients with advanced non-small cell lung cancer

**DOI:** 10.37349/etat.2023.00172

**Published:** 2023-10-11

**Authors:** Abhishek Mahajan, Devendra Goyal, Ujjwal Agarwal, Vijay Patil, Shreya Shukla, Vanita Noronha, Amit Joshi, Nandini Menon, Kumar Prabhash

**Affiliations:** Istituto Nazionale Tumori-IRCCS-Fondazione G. Pascale, Italy; ^1^Clatterbridge Centre for Oncology NHS Foundation Trust, L7 8YA Liverpool, UK; ^2^Department of Radiodiagnosis, Tata Memorial Hospital, Parel, Mumbai 400012, Maharashtra, India; ^3^Department of Medical Oncology, Tata Memorial Hospital, Parel, Mumbai 400012, Maharashtra, India

**Keywords:** Sarcopenia, advanced lung cancer inflammation index, modified advanced lung cancer inflammation index, advanced non-small cell lung cancer

## Abstract

**Aim:**

Sarcopenia and skeletal muscle density (SMD) have been shown to be both predictive and prognostic marker in oncology. Advanced lung cancer inflammation index (ALI) has been shown to predict overall survival (OS) in small cell lung cancer (SCLC). Computed tomography (CT) enables skeletal muscle to be quantified, whereas body mass index (BMI) cannot accurately reflect body composition. The purpose was to evaluate the prognostic value of modified ALI (mALI) using CT-determined third lumbar vertebra (L3) muscle index beyond original ALI and see the interaction between sarcopenia, SMD, neutrophil-lymphocyte ratio (NLR), ALI and mALI at baseline and post 4 cycles of chemotherapy and their effects on OS and progress free survival (PFS) in patients with advanced non-SCLC (NSCLC).

**Methods:**

This retrospective study consisted of a total of 285 advanced NSCLC patients. The morphometric parameters such as SMD, skeletal muscle index (SMI) and fat-free mass (FFM) were measured by CT at the L3 vertebra. ALI was defined as BMI × serum albumin/NLR and mALI was defined as SMI × serum albumin/NLR.

**Results:**

Sarcopenia was observed in over 70% of patients across all BMI categories. Patients having sarcopenia suffered from a higher incidence of chemotherapeutic drug toxicities but this was not found to be statistically significant. Concordance was seen between ALI and mALI in the pre-treatment setting and this was statistically significant. A significant proportion of patients with poor ALI (90.9%), poor pre-chemotherapy mALI (91.3%) and poor post-chemotherapy mALI (89%) had poor NLR and each of them was statistically significant.

**Conclusions:**

In both univariate and multivariate analyses, this study demonstrated the statistical significance of sarcopenia, SMD, and mALI as predictive factors for OS. Additionally, sarcopenia and SMD were also found to be statistically significant factors in predicting PFS. These biomarkers could potentially help triage patients for active nutritional intervention for better outcomes.

## Introduction

Humans have been interested the composition of the body since time immemorial. Body composition has been found to be a critical factor in cancer patients affecting chemotherapeutic efficacy and toxicity [1]. Previously understanding of cancer cachexia focused primarily on loss of body weight [2]. Loss of weight has remained an indicator of malnutrition and cachexia [3]. It is still used as an inclusion criterion and a principal endpoint in randomized clinical trials for various forms of cachexia treatment, including nutritional support [4, 5].

Sarcopenia is described as a loss of muscle mass and function. It is known to increase morbidity, healthcare costs, and mortality in the elderly. Sarcopenia affects 50–90% of untreated cancer patients and was shown to be a predictor of severe toxicity of patients included in phase 1 trials, suggesting that it should be considered an inclusion criterion for such studies [6, 7]. These patients had low performance status, shorter survival, more chemotherapy toxicities and post-operative infections, and longer post-operative hospitalization times [3, 4]. This can be explained by variances in drug pharmacokinetics when employing body surface area (BSA) for dose calculation. Patients with low lean body mass have a low volume of drug distribution and are frequently overdosed. Alterations in body composition (namely fat and muscle) occur in patients with cancer and can be associated with clinical outcomes. Patients undergo routine tests with high resolution diagnostic imaging. However, the information content of these images is barely exploited, owing to the lack of deployment of relevant methods and concepts [8].

The prognostic significance of advanced lung cancer inflammation index (ALI; a systemic inflammation-based index), which was the product of body mass index (BMI, kg/m^2^) and the serum albumin/neutrophil-lymphocyte ratio (NLR) have been shown. The results obtained showed a low ALI was significantly associated with poor prognosis in small cell lung cancer (SCLC) as well as non-SCLC (NSCLC) [9]. However, the interaction of ALI through the cycles of chemotherapy has never been explored.

Computed tomography (CT) provides an objective and reproducible means of quantifying skeletal muscle mass. A single cross-sectional area of muscle at the third lumbar vertebra (L3) is regarded as the gold standard for quantifying total-body skeletal muscle mass. BMI measurement, on the other hand, is limited in terms of evaluating sarcopenic status, because body weights do not precisely reflect body compositions. Weight loss is obscured in patients with a large tumor mass or collected fluid, such as, pleural effusion or body oedema [10].

High-quality images were used, which were obtained during the routine management of the patients, to provide accurate and practical studies of body composition across the cancer trajectory.

The purpose of the study is to evaluate the role of CT-based body composition measurement, in both pre-treatment and post-treatment (chemotherapy) setting of NSCLCs, in predicting clinical outcomes. The immediate and long-term effect of the presence of sarcopenia in patients with NSCLC and to investigate its clinical implications. Additionally, the study delved further to see if a sarcopenia based modified ALI (mALI) is better than then conventional BMI based ALI in predicting patient outcomes.

## Materials and methods

### Patients

This was a retrospective study in which data of 285 patients having baseline imaging positron emission tomography (PET)-CT or contrast enhanced (CECT) as well who underwent treatment for NSCLC at the institute was reviewed. The medical records and relevant data like demographics, tumour location, histopathology, pre-treatment staging, type of chemotherapy received, Response Evaluation Criteria in Solid Tumours (RECIST) response were obtained from a prospectively managed institutional database. The institutional picture archiving and communication system (PACS) system was used for retrieval of baseline and post treatment imaging in the form of either PET-CT or CECT scans. Electronic medical records and tumor registry was used for recurrence and survival information. Telephonic conversation was done for patients who were lost to follow up and survival information was inferred based on the same.

### Anthropometry and body composition

The BMI [weight/height^2^ (kg/m^2^)] and BSA [([height (cm) × weight (kg)]/3,600)^1/2^; m^2^] were calculated using height and weight data documented during patients initial physical assessment.

The established World Health Organization cut-offs in BMI were used to classify patients as:

(1). Underweight: BMI less than 18.5.
(2). Normal: BMI 18.5–24.9.
(3). Overweight: BMI 25–29.9.
(4). Obesity: BMI greater than or equal to 30 [11].

The processing of a CT scan and CT component of PET scans was done using Siemens Somatom Syngo viewer using a semi-automated algorithms program. A single observer determined the regional cross-sectional area of adipose tissue mass as well as the subcutaneous muscle mass at the L3 level. The mean mass was calculated using the average value of 3 consequent slices.

**Figure 1:**
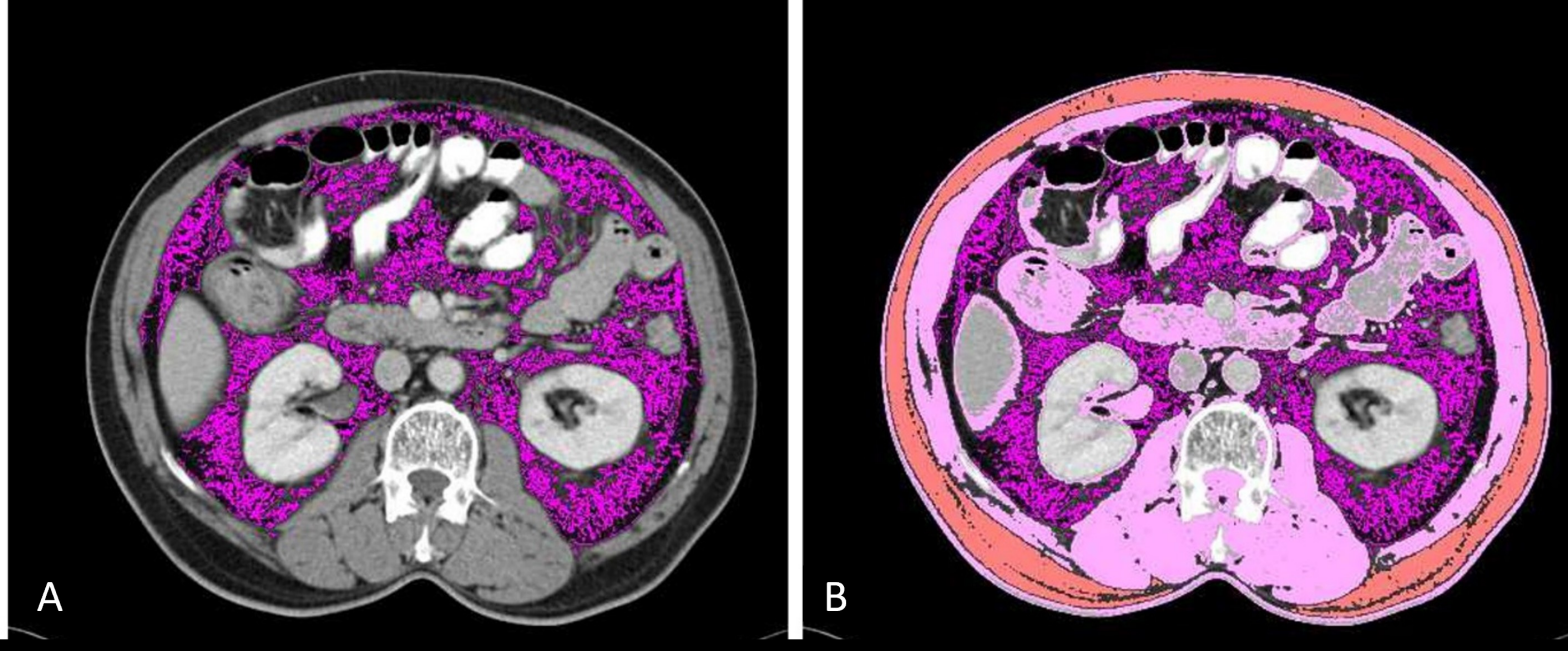
Demarcation of compartments. (A) Visceral fat is selected using a semi-automated technique by using appropriate HU as described in the text above (lavender); (B) skeletal muscle (pink) and subcutaneous fat (orange) are selected using a semi-automated technique by using appropriate HU as described in the text above. HU: Hounsfield unit

First, the L3 vertebral level in each scan was identified, followed by selection of the individual slice at the level of its transverse process. The visceral compartment deep to the abdominal muscles was demarcated next, followed by the subcutaneous muscle compartment and abdominal wall muscles. These steps were completed in a semi-automated fashion using programmed algorithms (Figure 1).

Total cross-sectional area of subcutaneous fat, visceral fat and subcutaneous muscle mass areas (cm^2^) was computed as a summation of the enclosed regions.

Tissue HU thresholds used for the computation were as follows:

(1). −29 to +150 for skeletal muscle.
(2). −190 to −30 for subcutaneous and intramuscular adipose tissue.
(3). −150 to −50 for visceral adipose tissue.

The skeletal muscle density (SMD) was calculated in HU within the paraspinal muscle compartment. Higher amounts of fatty infiltration were reflected as lower HU values [12].

### Calculations

Regression equations were used to derive total body fat mass (FM) and fat-free mass (FFM) from the total adipose tissue and total skeletal muscle mass respectively.

Visceral to subcutaneous fat tissue ratio and subcutaneous fat to skeletal muscle ratio were also calculated.

Total L3 skeletal muscle cross-sectional area (cm^2^) was normalized for stature and expressed in units of cm^2^/m^2^.

**Figure 2:**
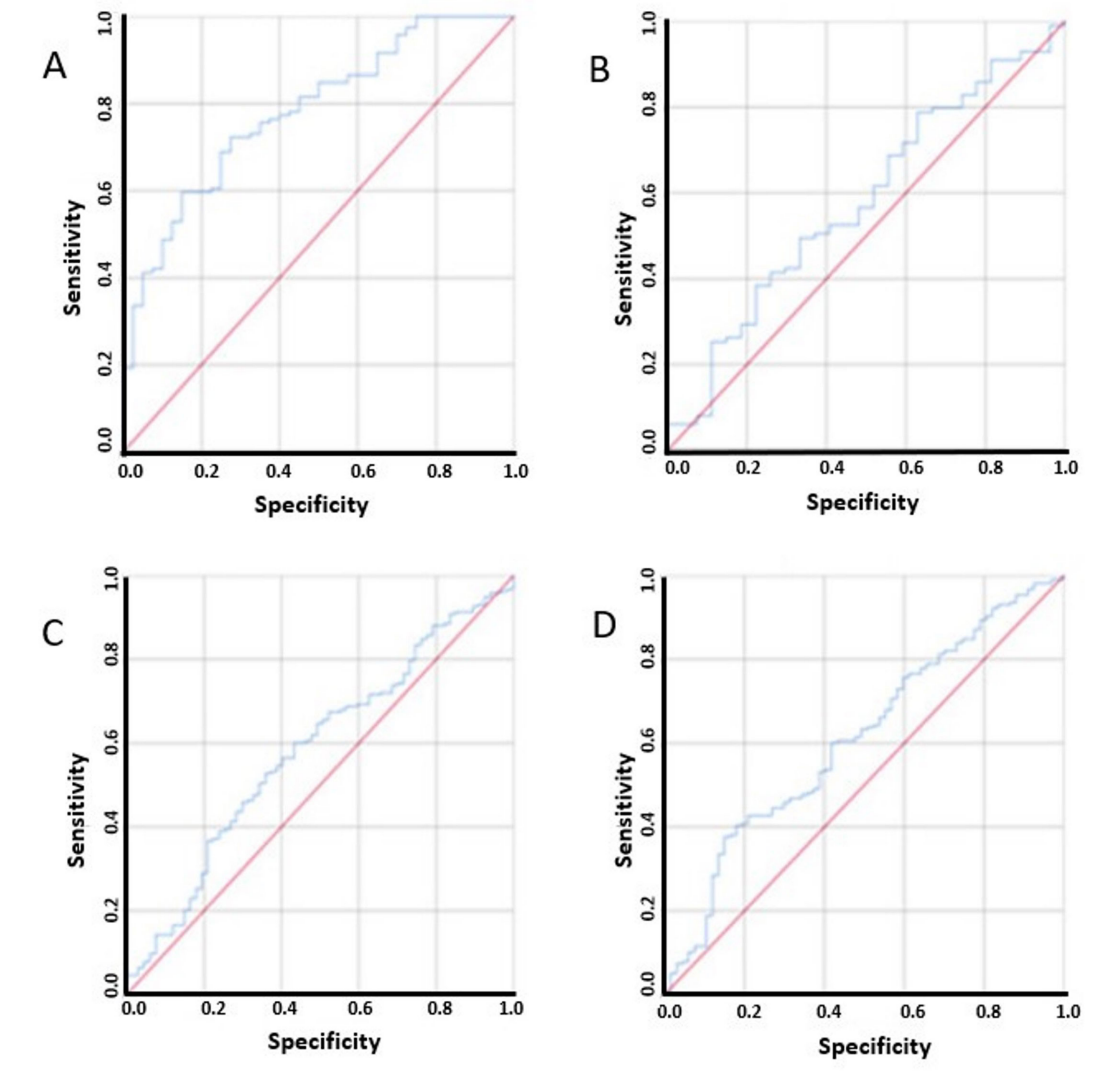
ROC curves used to determine cut-off for sarcopenia in males, females, ALI and mALI. (A) ROC curve used to determine cut-off for sarcopenia in males; (B) ROC curve used to determine cut-off for sarcopenia in females; (C) ROC curve used to determine cut-off for ALI; (D) ROC curve used to determine cut-off for mALI

The cut-off for sarcopenia has been shown previously to vary among different ethnic population. A receiver operator characteristic (ROC) was drawn to determine the appropriate sex-specific cut-off for the population. A value of 49.32 cm^2^/m^2^ in males showed a sensitivity of 72% and specificity of 70% in predicting sarcopenia (Figure 2). A similar ROC was drawn for females and a value of 41.23 cm^2^/m^2^ showed a sensitivity of 68% and specificity of 45% in predicting sarcopenic (Figure 2). A log-rank chi-square test (χ²) statistic was done to compare cut-offs of the study with the ones originally used by Prado et al. [1]: 52.4 cm^2^/m^2^ for males and 38.5 cm^2^/m^2^ for females. Statistical significance was found with both set of values and due to the established nature of the cut-offs described by Prado et al. [1] and it was decided to use the same cut-offs to allow comprehensive comparison.

ROC curves were also used to determine the cut-offs for ALI and mALI due to inconsistent nature of the values previously used in literature [9, 13–17]. For ALI, a value 28.02 of showed a sensitivity of 65% and specificity of 50%, while for mALI a value of 55.33 showed a sensitivity of 63% and specificity of 50% (Figure 2). A cut-off of 36.02 was determined for defined good (> 36.62) and poor (≤ 36.62) SMD.

NLR was defined a good (≤ 3) and poor (> 3) according to the cut-offs defined by Diem et al. [18]. Post chemotherapeutic weight was not available so BMI and ALI were not calculated in the post treatment stage.

### Statistical analysis

Statistical Package for Social Sciences (SPSS) 21 statistical package (IBM) was used for data analysis. The results were expressed as mean  ±  standard deviation (SD). Fisher’s exact test was used to compare morpho-metrics related to patients’ anthropomorphic measurements, disease characteristics, and pathological features, while analysis of variance techniques was used when the number of groups exceeded two. Kaplan-Meier method was used to detect associations between body composition parameters and overall survival (OS) or progression free survival (PFS) and compared with the log-rank test.

Significant univariate variables were included in a multivariate logistic regression analysis which was done by Cox proportional hazard regression models to assess the association of body composition measurements for significant patient characteristics. For all statistical tests, *P*-values ≤ 0.05 were considered significant.

## Results

### Study population characteristics

Body compositions were analysed for the 285 lung cancer patients. Out of the total study population, the majority consisted of males (*n* = 159, 56%), with a mean age of 53.8 years. All patients shared a histopathological diagnosis of adenocarcinoma (100%). Among the entire cohort, 59 (20.7%) individuals were identified as smokers, of whom 54 (91.5%) were males.

At baseline, 217 (76.2%) patients had a BMI < 25, 61 (21.4%) had a BMI 25–30 and 7 (2.4%) had a BMI > 30.

Based on the cut-off of < 36.63 HU as poor muscle quality, 185 (64.9%) had poor muscle quality. The mean SMD in our study population was 26.89 HU with statistical significance between males and females (*P* = 0).

At baseline, 175 (61.4%) patients had a poor (> 3) NLR with no statistical difference between males and females.

### Body mass composition as a predictor of outcomes

The interactions between morphometrics and clinical parameters are demonstrated in Tables 1 and 2.

**Table 1:** Comparison of clinico-pathological features between patients with and without sarcopenia

**Parameter**		**Total**	**Pre-chemotherapy sarcopenia**		**Total**	**Post-chemotherapy sarcopenia**		***P*-value (sex)**	
			**Yes**	**No**		**Yes**	**No**	**Baseline**	**Post-chemotherapy**
BMI	< 18.5%	285	42	14	-	-	-	**0**	-
	18.5–24.99%		89	72					
	25.0–29.99%		26	35					
	≥ 30%		5	2					
Age	≤ 65 Years	285	141	111	274	150	95	0.40	0.12
	> 65 Years		21	12		22	7		
Smoker	Yes	285	41	18	274	43	11	**0.02**	**0**
	No		121	105		129	91		
SMD	Good (> 36.62)	285	34	66	274	42	59	**0**	**0**
	Poor (≤ 36.62)		128	57		130	43		
Gender	Male	285	115	44	274	121	29	**0**	**0**
	Female		47	79		51	73		
ALI	Good (> 28.02)	285	55	53	274	-	-	0.12	-
	Poor (≤ 28.02)		107	70					
mALI	Good (> 55.33)	285	54	58	274	80	67	**0.02**	**0.02**
	Poor (≤ 55.33)		108	65		92	35		
NLR	Good (≤ 3)	285	55	55	274	88	63	0.06	0.08
	Poor (> 3)		107	68		84	39		
Chemotherapy regimen received	Geftinib	285	78	65	-	-	-	0.43	-
	Pemetrexed plus platins		84	58					
Grades of drug toxicity	High grade	273	87	83	-	-	-	0.06	-
	Low grade		65	38					
Performance status	0–1	285	150	118	-	161	97	0.23	0.61
	≥ 2		12	5		11	5		
Progression	Yes	285	118	92		-	-	0.71	
	No		44	31					
Final status	Alive	285	25	42	-	-	-	**0**	-
	Dead		137	81	-	-	-	-	-

-: not applicable. Bold values indicate significant *P*-values

**Table 2:** Comparison of clinico-pathological features between patients with good and poor SMD at baseline

**Parameter**		**Total**	**Pre-chemotherapy SMD**		**Total**	**Post-chemotherapy SMD**		***P*-value (sex)**	
			**Good (> 36.62)**	**Poor (≤ 36.62)**		**Good (> 36.62)**	**Poor (≤ 36.62)**	**Baseline**	**Post-chemotherapy**
BMI	< 18.5%	285	14	42	-	-	-	0.317	-
	18.5–24.99%		62	99					
	25.0–29.99%		21	40					
	≥ 30%		3	4					
Smoker	Yes	285	18	41	274	18	35	0.408	-
	No		82	144		80	142		
Age	≤ 65 Years	285	90	162	274	88	159	0.540	0.297
	> 65 Years		10	23		7	20		
mALI	Good (> 55.33)	285	47	65	274	63	84	0.05	**0.03**
	Poor (≤ 55.33)		53	120		38	89		
Gender	Male	285	60	99	274	58	101	0.293	0.680
	Female		40	86		43	83		
ALI	Good (> 28.02)	285	42	66	-	-	-	0.294	-
	Poor (≤ 28.02)		58	119					
Sarcopenia	Yes	285	34	128	274	42	130	**0**	**0**
	No		66	57		59	43		
NLR	Good (≤ 3)	285	41	69	274	57	94	0.54	0.74
	Poor (> 3)		59	116		44	79		
Chemotherapy regimen received	Geftinib	285	50	93	-	-	-	0.96	-
	Pemetrexed plus platins		50	92					
Grades of drug toxicity	High grade	273	62	108	-	-	-	0.80	-
	Low grade		36	67					
Performance status	0–1	285	98	170	-	-	-	0.07	-
	≥ 2		2	15					
Progression	Yes	285	84	126	-	-	-	0.68	-
	No		28	47					
Final status	Alive	285	37	30	-	-	-	**0**	-
	Dead		63	155	--	-	-	-	-

-: not applicable. Bold values indicate significant *P*-values

The study population exhibited a mean sarcopenia index of 44.4 cm²/m². Notably, a statistically significant distinction was observed between males (48.26 cm^2^/m^2^) and females (39.63 cm^2^/m^2^) *P* = 0.01. Sarcopenia was prevalent in the overall population, accounting for 162 (56.8%) individuals. Among the elderly subgroup (aged over 65), 63.63% (21 out of 33) displayed sarcopenia, while in individuals under 65, 55.95% (141 out of 252) were identified as sarcopenic. No statistical significance, however, was identified between these two subgroups.

Sarcopenic patients exhibited a slightly higher mean age of 54.5 years compared to 52.9 years for non-sarcopenic individuals. The male population predominantly showed sarcopenia (72.3% males *vs.* 37.3% females; *P* = 0).

A higher proportion of smokers were found amongst sarcopenics (25.3%) as *vs.* non-sarcopenics (17.1%) and this was found to be significant with a *P* = 0.02.

The patients were divided into two groups according to sarcopenic index. The mean BMI was found to be nearly equal in patients with and without sarcopenia (21.5 kg/m^2^ *vs.* 22.9 kg/m^2^). As expected, 75% (*n* = 42) of patients with low BMI (< 18.5, *n* = 56) were sarcopenics, but 72.5% of patients (*n* = 161) with a BMI range of 18.5–29.99 were also sarcopenics. Even in patients with BMI > 30, 71.4% patients (*n* = 5) were sarcopenics, signifying sarcopenic obesity. Hence, a remarkably consistent proportion exceeding 70% of patients exhibited sarcopenia across all BMI categories, and this finding yielded significant with a *P* = 0. The correlation between SMD and sarcopenia was evident, as 79% of sarcopenic patients displayed poor SMD compared to 46% among non-sarcopenic patients. This association remained consistent in both the pre-chemotherapy and post-chemotherapy scenarios, demonstrating statistical significance with a *P* = 0 for both pre and post-treatment situations.

Based on the cut-off of ≤ 28.02 for poor ALI, patients were divided into two groups and compared with sarcopenia. Sixty six percent (66%) sarcopenic patients had poor ALI *vs.* 56.9% amongst non-sarcopenics, but this difference was not found to be statistically significant.

Similar division was done for mALI with a cut-off of 55.33 and 66.67% sarcopenic patients had poor mALI as compared to 52.8% amongst non-sarcopenics, and this decreased to 53.4% and 34.3% respectively post-chemotherapy. Statistical significance was seen between mALI and sarcopenia in both the pre and post treatment settings with *P* = 0.02.

Patients with sarcopenia experienced a greater occurrence of chemotherapeutic drug toxicities, totaling 152 cases as opposed to 121 in non-sarcopenic individuals. However, this disparity did not yield statistical significance. Statistically significant differences were also seen between sarcopenia and FFM index.

### Interaction of ALI and mALI over course of treatment

The interactions between ALI and mALI are demonstrated in Tables 3 and 4.

**Table 3:** Comparison of clinico-pathological features between patients with good and poor ALI

**Parameter**		**Total**	**ALI**		***P*-value**
			**Good (> 28.02)**	**Poor (≤ 28.02)**	
BMI	< 18.5%	285	9	47	**0**
	18.5–24.99%		59	102	
	25.0–29.99%		34	27	
	≥ 30%		6	1	
Smoker	Yes	285	22	37	0.91
	No		86	140	
Age	≤ 65 Years	285	94	158	0.57
	> 65 Years		14	19	
SMD	Good (> 36.62)	285	42	58	0.29
	Poor (≤ 36.62)		66	119	
Gender	Male	285	55	104	0.20
	Female		53	73	
Sarcopenia	Yes	285	55	107	0.12
	No		53	70	
mALI	Good (> 55.33)	285	95	17	**0**
	Poor (≤ 55.33)		13	160	
NLR	Good (≤ 3)	285	94	16	**0**
	Poor (> 3)		14	161	
Chemotherapy regimen received	Geftinib	285	51	92	0.44
	Pemetrexed plus platins		57	85	
Grades of drug toxicity	High grade	273	61	109	0.62
	Low grade		40	63	
Performance status	0–1	285	103	165	0.46
	≥ 2		5	12	
Progression	Yes	285	78	132	0.66
	No		30	45	
Final status	Alive	285	33	34	**0.03**
	Dead		75	143	

Bold values indicate significant *P*-values

**Table 4:** Comparison of clinico-pathological features between patients with good and poor mALI

**Parameter**		**Total**	**Pre-chemotherapy mALI**		**Total**	**Post-chemotherapy mALI**		***P*-value (sex)**	
			**Good (> 55.33)**	**Poor (≤ 55.33)**		**Good (> 55.33)**	**Poor (≤ 55.33)**	**Baseline**	**Post-chemotherapy**
BMI	< 18.5%	285	17	39	-	-	-	0.17	-
	18.5–24.99%		65	96					
	25.0–29.99%		25	36					
	≥ 30%		5	2					
Smoker	Yes	285	26	33	274	30	24	0.40	0.75
	No		86	140		117	103		
Age	≤ 65 Years	285	96	156	274	136	109	0.25	0.07
	> 65 Years		16	17		11	18		
SMD	Good (> 36.62)	285	47	53	274	63	38	0.05	**0.03**
	Poor (≤ 36.62)		65	120		84	89		
Gender	Male	285	67	92	274	88	62	0.27	0.07
	Female		45	81		59	65		
ALI	Good (> 28.02)	285	95	13	-	-	-	**0**	-
	Poor (≤ 28.02)		17	160					
Sarcopenia	Yes	285	54	108	274	80	92	**0.02**	**0**
	No		58	65		67	35		
NLR	Good (≤ 3)	285	95	15	274	137	14	**0**	**0**
	Poor (> 3)		17	158		10	113		
Chemotherapy regimen received	Geftinib	285	49	94	-	-	-	0.08	-
	Pemetrexed plus platins		63	79					
Grades of drug toxicity	High grade	273	66	104	-	-	-	0.74	-
	Low grade		39	64					
Performance status	0–1	285	104	164	274	138	120	0.50	0.83
	≥ 2		8	9		9	7		
Progression	Yes	285	84	126	-	-	-	0.68	-
	No		28	47					
Final status	Alive	285	33	34	-	-	-	0.06	-
	Dead		79	139					

-: not applicable. Bold values indicate significant *P*-values

The patients were divided into two groups according to ALI and mALI using a cut-off ≤ 28.02 and ≤ 55.33 respectively. The average ALI for the entire population was 26.10, while the mean mALI was 52.05. Statistical significance was observed in ALI between males and females, whereas such significance was not evident in mALI. Among the patients, 83.9% of those with a BMI less than 18.5, 58.1% with a BMI ranging from 18.5 to 29.99, and 14.2% with a BMI exceeding 30 exhibited poor ALI. This observation was deemed statistically significant with a *P* = 0. No significance was seen between mALI and BMI.

A concordance was observed between ALI and mALI in the pre-treatment context, where 90% of patients displaying poor ALI also exhibited poor mALI. This concordance held statistical significance, evident by a *P* = 0. In the post treatment setting poor SMD was more prevalent in patients with poor mALI (70%) *vs.* good mALI (57%) and this was statistically significant with a *P* = 0.03. SMD did not correlate with ALI or mALI in the pre-treatment setting.

mALI and sarcopenia showed concordance and statistical significance in both the pre and post treatment setting, but this was not seen with ALI.

A substantial proportion of patients demonstrated a correlation between poor ALI, poor pre-chemotherapy mALI, and poor post-chemotherapy mALI with poor NLR, with percentages of 90.9%, 91.3%, and 89% respectively. Each of these associations exhibited statistical significance. Patients with good and poor ALI, pre-chemotherapy and post-chemotherapy mALI were otherwise similar with respect to age, gender, chemotherapeutic regimen received, grades of chemo-toxicity and performance status.

### Survival

The Univariate and Multivariate Survival analysis are demonstrated in Table 5.

**Table 5:** Univariate and multivariate analyses of possible risk factors for OS and PFS by Cox proportional hazards model

**Parameter**	**OS univariate *P*-value**	**OS multivariate *P*-value**	**PFS univariate *P*-value**	**PFS multivariate *P*-value**
Age	**0.01**	0.086	0.331	-
Smoking history	**0.01**	0.533	0.501	-
BMI	**0.007**	0.267	0.215	-
Pre-chemotherapy sarcoepnia	**0**	**0**	**0**	0.446
Pre-chemotherapy SMD	**0.005**	0.132	**0.001**	0.781
Pre-chemotherapy ALI	**0.033**	0.45	0.377	-
Pre-chemotherapy mALI	**0.012**	0.135	0.414	-
Pre-chemotherapy NLR	0.148	-	0.208	-
Post-chemotherapy sarcoepnia	**0**	**0.001**	**0**	**0.001**
Post-chemotherapy SMD	**0.001**	**0.047**	**0**	0.292
Post-chemotherapy mALI	**0**	**0.034**	0.077	-
Post-chemotherapy NLR	**0.022**	0.418	0.348	-

-: not applicable. Bold values indicate significant *P*-values

To study whether body mass composition may be related to tumor biology in lung cancer, the study examined both morphometric and clinical parameters in respect to outcomes. The mean follow-up was 54 months, during which time a total of 210 (73.6%) patients experienced progression and 218 (76.4%) patients died.

The mean OS was 20.97 months with no statistical difference between male and female. The mean OS in sarcopenic patients were 14.69 months and in non-sarcopenic patients were 29.27 months with significant statistical difference in both the pre-chemotherapeutic and post-chemotherapeutic setting in univariate analysis (*P* = 0) and multivariate analysis (*P* = 0).

The mean OS in patients with good ALI was 22.68 months and in patients with poor ALI were 19.94 months with significant statistical difference in univariate analysis (*P* = 0.03) and but not in multivariate analysis (*P* = 0.45).

The mean OS in patients with good mALI were 23.59 months and in patients with poor mALI were 19.29 months with significant statistical difference in both the pre-chemotherapeutic and post-chemotherapeutic setting in univariate analysis (*P* = 0) and only in post-chemotherapeutic setting in multivariate analysis (*P* = 0.034).

OS was found to be statistically significant in patients with respect to their pre-chemotherapeutic SMD and post-chemotherapeutic SMD in univariate analysis (*P* = 0.005 and *P* = 0.001) and post-chemotherapeutic SMD in the multivariate analysis (*P* = 0.047).

The difference in OS was found to be statistically significant in patients with respect to their age, smoking history, BMI and post-chemotherapeutic NLR on the univariate analysis (*P* = 0.01, 0.01, 0.007 and 0.02 respectively) but not in the multivariate analysis.

On univariate analysis none of the other morphometric and clinical parameters showed a trend toward a difference in OS.

Mean PFS was 7.59 months with no statistical difference between male and female. The mean PFS in sarcopenic patients were 5.86 months and in non-sarcopenic patients were 9.87 months with significant statistical difference in both the pre-chemotherapeutic and post-chemotherapeutic setting in univariate analysis (*P* = 0) and only the post-chemotherapeutic setting in multivariate analysis (*P* = 0).

PFS was found to be statistically significant in patients with respect to their pre-chemotherapeutic SMD and post-chemotherapeutic SMD in univariate analysis (*P* = 0.001 and *P* = 0) but not in the multivariate analysis (*P* = 0.047).

The mean PFS in patients with good ALI was 7.94 months and in patients with poor ALI were 7.38 months with no significant statistical difference in univariate analysis (*P* = 0.249).

The mean PFS in patients with good mALI was 8.14 months and in patients with poor mALI was 7.24 months with no significant statistical difference in univariate analysis (*P* = 0.293).

On univariate analysis, none of the other morphometric and clinical parameters showed a trend toward a difference in PFS.

## Discussion

**Figure 3:**
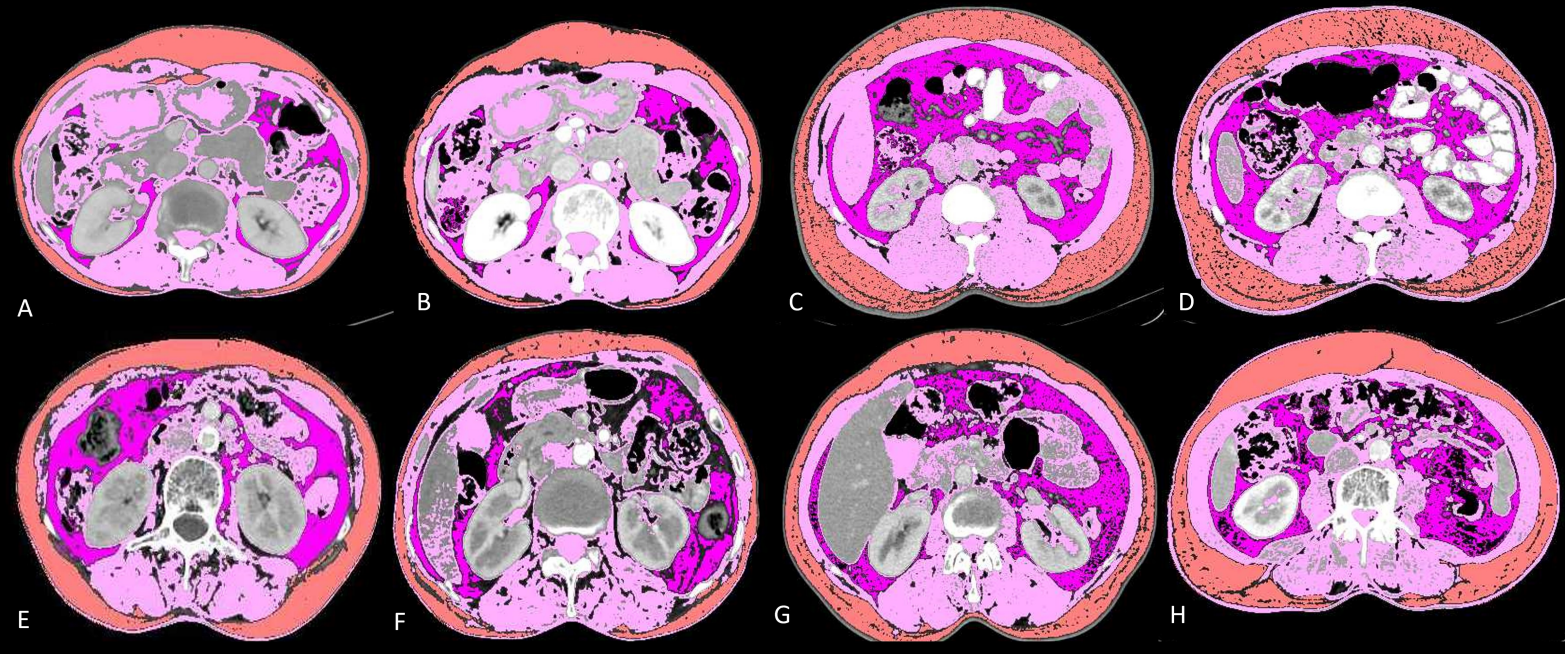
Baseline and post-chemotherapy morphometric analysis in 4 cases showing interaction between different parameters. Details below: visceral fat (lavender); skeletal muscle (pink) and subcutaneous fat (orange)

This was a large-scale, single-institution study where sarcopenia, NLR, ALI and mALI were assessed as potential novel risk factors for clinical outcomes in 285 patients with advanced NSCLC. The findings are best reflected in the 4-case capsule presented in Figure 3. Body compositions were analysed on CT for 285 lung cancer patients. The majority of patients were male and the mean age was 53.8 years. Histopathology of all the patients was adenocarcinoma (100%). Fifty nine (20.7%) of total patients were smokers, out of which 54 (91.5%) were males.

### Case capsule 1

It shows baseline and post-chemotherapy morphometric analysis of a 65-year-old lady with stage IV adenocarcinoma lung (Figure 3A and 3B). She had a baseline skeletal muscle index (SMI) of 44.63, SMD of 38.82, NLR of 2.94, ALI of 34.41 and mALI of 62.02 while in the post chemotherapeutic setting her SMI was 41.84, SMD was 38.48, NLR was 2.08 and mALI was 74.16. Her OS was 33.23 months which was higher than the mean OS which is consistent with her non sarcopenic, good SMD, good ALI, good NLR and good mALI status.

### Case capsule 2

It shows baseline and post-chemotherapy morphometric analysis of a 56-year-old man with stage III adenocarcinoma lung (Figure 3C and 3D). He had a baseline SMI of 60.15, SMD of 30.56, NLR of 4.73, ALI of 20.58 and mALI of 55.92 while in the post chemotherapeutic setting his SMI was 59.01, SMD was 37.06, NLR was 3.2 and mALI was 73.75. At baseline he was non sarcopenic but with poor SMD, poor NLR, poor ALI and borderline normal mALI. In the post chemotherapeutic setting he was persistently non-sarcopenic with improvements in his SMD and NLR which ultimately reflected in his OS which were 30.80 months which was higher than the mean OS of males which was 20.02 months.

### Case capsule 3

It shows baseline and post-chemotherapy morphometric analysis of a 52-year-old lady with stage IV adenocarcinoma lung (Figure 3E and 3F). She had a baseline SMI of 36.52, SMD of 9.97, NLR of 3.78, ALI of 27.83 and mALI of 39.53 while in the post chemotherapeutic setting her SMI was 29.55, SMD was 2.3, NLR was 2.42 and mALI was 38.2. At baseline she was sarcopenic, had poor SMD, NLR, ALI and mALI. In the post chemotherapeutic setting her sarcopenia, SMD and mALI worsened while her NLR improved. Her OS was 4 months which is well below the mean OS for females (22.19 months) thus reflecting that sarcopenia, SMD and mALI are independent factors for predicting survival as reflected in the multivariate analysis.

### Case capsule 4

It shows baseline and post-chemotherapy morphometric analysis of a 40-year-old man with stage IV adenocarcinoma lung (Figure 3G and 3H). He had a baseline SMI of 44.15, SMD of 10.50, NLR of 5.35, ALI of 12.56 and mALI of 27.43 while in the post chemotherapeutic setting his SMI was 41.88, SMD was 4.15, NLR was 6.56 and mALI was 24.24. At baseline he was sarcopenic with poor SMD, NLR, ALI and mALI. In the post chemotherapeutic setting all the parameters worsened. Deeper analysis revealed that there was severe cancer associated inflammation as evident with persistently high NLR. The subcutaneous fat area increased from 116.06 at baseline to 238.71 in the post chemotherapeutic setting. There was associated fatty infiltration of the muscle as revealed by a decrease in SMD from 10.5 to 4.15. All these reflected in his OS which was 6.07 months well below the mean OS of males which was 20.02 months.

The mean SMD in our study population was 26.89 HU with statistical significance between males and females (*P* = 0). Based on the cut-off of < 36.62 HU as poor muscle quality, 200 (67.7%) had poor muscle quality. The mean sarcopenia index in our population was 44.4 cm^2^/m^2^. There was a statistically significant difference in sarcopenia index between males and females. The overall prevalence of sarcopenia within the population was 162 (56.8%). These patients were slightly older (mean age of 54.5 years *vs.* 52.9 years) and sarcopenia was predominantly seen in the male population (72.3% males *vs.* 37.3% females; *P* < 0).

A nearly consistent proportion of more than 70% patients were found to be suffering from sarcopenia across all the BMI categories and this was found to be significant with a *P* = 0. In the group of sarcopenic patients, 79% displayed poor SMD, whereas among non-sarcopenic patients, this figure was 46%. This relation held true in both the pre-chemotherapy and post-chemotherapy setting and was statistically significant with *P* = 0 in both pre and post treatment settings. Patients having sarcopenia suffered from a higher incidence of chemotherapeutic drug toxicities 152 as compared to 121 amongst non-sarcopenics, but this was not found to be statistically significant.

The mean ALI of the population was 26.10 and that of mALI was 52.05 with statistical significance between male and female in ALI but not mALI. Concordance was seen between ALI and mALI in the pre-treatment setting with 90% of patients with poor ALI having poor mALI and this was statistically significant with a *P* = 0.

mALI and sarcopenia showed concordance and statistical significance in both the pre and post treatment setting, but this was not seen with ALI. Significantly, 90.9% of patients with poor ALI, 91.3% with poor pre-chemotherapy mALI, and 89% with poor post-chemotherapy mALI also exhibited poor NLR. Only a single published study is available which has delved into the relationship between mALI and ALI. This study has 89.4% concordance between patients with good and bad ALI and mALI, *vs.* 92.4% seen in study by Kim et al. [15].

In this study patients with good mALI had higher OS compared with patients with poor mALI (23.59 months compared to 19.29 months) and this was found to be statistically significant in both the pre-chemotherapeutic and post-chemotherapeutic setting in univariate analysis (*P* = 0) and only in post-chemotherapeutic setting in multivariate analysis (*P* = 0.034).

Patients with good ALI had higher OS compared with patients with poor ALI (22.68 months compared to 19.94 months). This was found to have significant statistical difference in univariate analysis (*P* = 0.03) and but not in multivariate analysis (*P* = 0.45).

Kim et al. [15] observed that patients with poor ALI had shorter OS than patients with good ALI (median, 6.8 months *vs.* 15.8 months; *P* < 0.001), and patients with poor mALI had shorter OS than patients with good mALI (median, 6.8 months *vs.* 16.5 months; *P* < 0.001). There was no significant difference in estimates of median survival time between poor ALI and poor mALI (*Z* = 0, *P* = 1.000) and between good ALI and good mALI. But this study was done in patients with SCLC, while the study focused exclusively on patients with advanced adenocarcinoma. Buentzel et al. [19] showed that muscle loss, regardless of cancer stage, is an independent risk factor for increased death risk in lung cancer patients. Another study by Yang et al. [20] showed that sarcopenia was associated with a shorter OS in patients with lung cancer and this association existed for both NSCLC and SCLC. Early detection of sarcopenia and adequate treatments are evidently essential due to the prognostic relevance of sarcopenia in cancer patients [21]. Four exercise strategies, including resistance training, resistance training plus nutritional supplementation, multimodal exercise programmes and blood flow restriction training are currently used to treat sarcopenia [22]. Nutritional intervention in the form of protein supplementation, essential amino acids supplementation, β-hydroxy-β-methylbutyrate (HMB) supplementation, Vitamin D supplementation and creatine supplementation combined with exercise showed greater improvement in muscle strength [22, 23]. Aerobic exercise, including voluntary wheel running, treadmill exercise, dance, cycle ergometer and bicycle training lead to increase in muscle size, muscle strength, grip strength and increase quadriceps volume [24, 25]. Resistance exercise such as stretch-shortening contraction, resistance wheel exercise, climbing a 1–3 vertical ladder and progressive resistance exercise training lead to increase in tibialis anterior and soleus muscle mass and muscle power and function [26, 27]. The postoperative complication rate was increased in patients with sarcopenia in the meta-analysis by Nishimura et al. [28]. Two meta-analyses by Wang et al. [29] and Takenaka et al. [30] revealed a significantly worse disease control rate in sarcopenic *vs.* non-sarcopenic participants. In the meta-analyses by Wang et al. [29], Deng et al. [31], Lee et al. [32], and Takenaka et al. [30], pre-treatment sarcopenia was substantially associated to decreased progression-free survival rates in patients undergoing immunotherapy. Kawaguchi et al. [33] demonstrated shortened disease-free survival for sarcopenic patients. Up to 50% of individuals with lung cancer experience sarcopenia, a serious health risk. Due to the association to enhanced surgical complications, lower immunotherapy response rates, and increased mortality, its diagnosis in this population should not be taken lightly.

The present study has the following limitations which need consideration. Given the retrospective nature of the study, the correlation of sarcopenia and SMD with other metrics could not be assessed. Additionally due to the retrospective nature access to post-chemotherapeutic weight of the patients was and thus post-chemotherapeutic BMI and ALI could not be assessed. Hence, the study could only assess the interaction of ALI and mALI at the baseline and not the post treatment setting.

Summarising our study showed that sarcopenia, SMD and mALI were statistically significant factors in predicting OS in both univariate and multivariate analysis, while sarcopenia and SMD also were statistically significant factors in predicting PFS. Sarcopenia, sarcopenic obesity and visceral obesity may be associated with negative oncological outcomes. Imaging assessment of body composition can be readily applied in the clinical setting with the potential to improve individual nutritional care. This personalized cancer management strategy may reduce treatment-related toxicities and ultimately improve patient outcomes. Loss of muscle mass is a significant cause of morbidity in lung cancer patients. Loss of muscle mass and function may occur before cachexia does clinically, highlighting the significance of addressing sarcopenia rather than only looking at weight loss. Understanding this relationship and its associated factors will provide opportunities for focused intervention to improve clinical outcomes.

To conclude, this study highlighted the significant predictive role of sarcopenia, SMD, and mALI in both univariate and multivariate analyses for OS. Additionally, both sarcopenia and SMD were identified as statistically significant factors in predicting PFS. These biomarkers could potentially help triage patients for chemotherapy according to their tolerability and active nutritional intervention for better outcomes.

## Abbreviations

ALI: advanced lung cancer inflammation index
BMI: body mass index
CT: computed tomography
L3: third lumbar vertebra
mALI: modified advanced lung cancer inflammation index
NLR: neutrophil-lymphocyte ratio
NSCLC: non-small cell lung cancer
OS: overall survival
PET: positron emission tomography
PFS: progression free survival
SMD: skeletal muscle density
SMI: skeletal muscle index

## Declarations

### Availability of data and materials

The datasets generated in this study are available on request to Abhishek Mahajan (drabhishek.mahajan@yahoo.in; abhiradiology@gmail.com).

### Copyright

© The Author(s) 2023.
